# Preparation of Reswellable Amorphous Porous Celluloses through Hydrogelation from Ionic Liquid Solutions

**DOI:** 10.3390/ma12193249

**Published:** 2019-10-04

**Authors:** Satoshi Idenoue, Yoshitaka Oga, Daichi Hashimoto, Kazuya Yamamoto, Jun-ichi Kadokawa

**Affiliations:** Department of Chemistry, Biotechnology, and Chemical Engineering, Graduate School of Science and Engineering, Kagoshima University, 1-21-40 Korimoto, Kagoshima 860-0065, Japan; k0399304@kadai.jp (S.I.); k6306570@kadai.jp (Y.O.); k9233501@kadai.jp (D.H.); yamamoto@eng.kagoshima-u.ac.jp (K.Y.)

**Keywords:** amorphous, hydrogel, ionic liquid, regenerated cellulose, reswelling

## Abstract

In this study, we have performed the preparation of reswellable amorphous porous celluloses through regeneration from hydrogels. The cellulose hydrogels were first prepared from solutions with an ionic liquid, 1-butyl-3-methylimidazolium chloride (BMIMCl), in different concentrations. Lyophilization of the hydrogels efficiently produced the regenerated celluloses. The powder X-ray diffraction and scanning electron microscopic measurements of the products suggest an amorphous structure and porous morphology, respectively. Furthermore, the pore sizes of the regenerated celluloses, or in turn, the network sizes of cellulose chains in the hydrogels, were dependent on the concentrations of the initially prepared solutions with BMIMCl, which also affected the tensile mechanical properties. It was suggested that the dissolution states of the cellulose chains in the solutions were different, in accordance with the concentrations, which representatively dominated the pore and network sizes of the above materials. When the porous celluloses were immersed in water, reswelling was observed to regenerate the hydrogels.

## 1. Introduction

Cellulose is the most abundant organic substance on the earth, and accordingly, it is a very important biomass resource [1]. Because of the β(1→4)-linked glycosidic arrangement of glucose repeating units, cellulose forms highly crystalline structures with elongated stiff chain packing, which can function as a structural material in biological systems, such as in components in cell walls. Cellulose, therefore, is currently used as a fibrous material in practical applications, e.g., in textiles, clothes, and furniture. Apart from fibrils, to extend the application of cellulose to soft and plasticized materials, its stiff crystalline structure should be relaxed by incorporating some moieties among the cellulose chains. For example, cellulosic flexible materials can be fabricated by adding plasticizers, such as cellophane with glycerin [2]. A number of swelling approaches for cellulose have also been performed to provide soft materials, such as in the case of hydrogels [3,4,5,6]. In addition to their flexible properties, the acylation of hydroxy groups on cellulose chains is a useful method to exhibit thermoplasticity in cellulosic materials, such as in the practically used cellulose triacetate [7]. 

We have already found that a flexible cellulose film, fabricated through composition with an ionic liquid, namely 1-butyl-3-methylimidazolium chloride (BMIMCl), through ion gel formation, shows thermoplasticity and thermal processability without acylation [8]. Ionic liquids are molten salts which exhibit a liquid state at temperatures below the boiling point of water. Since BMIMCl was found to dissolve cellulose [9], ionic liquids have been identified as powerful solvents for cellulose [10,11,12,13,14,15,16,17,18]. We have also reported that that cellulose formed the ion gel with BMIMCl by standing a cellulose/BMIMCl solution at room temperature [19]. By adapting the method for the ion gel formation from cotton cellulose and BMIMCl, we have successfully fabricated the abovementioned thermoplastic cellulose film combined with BMIMCl. 

On the other hand, cellulose hydrogels were fabricated from solutions with ionic liquids, such as BMIMCl and 1-allyl-3-methylimidazolium chloride [20,21,22,23,24]. For example, Peng et al. have reported that a cellulose hydrogel was facilely obtained by soaking a 5 wt.% cellulose/BMIMCl solution in water [25]. Furthermore, this study investigated the detailed characterization and properties of the hydrogels. For example, the X-ray diffraction (XRD) result of the regenerated cellulose after lyophilization of the hydrogel indicated a remarkable decrease in the crystallinity of cellulose. The above previous studies have inspired us to potentially fabricate unique cellulosic materials with low crystallinity using BMIMCl. Consequently, in this paper, we would like to report the possible preparation of porous celluloses with mostly amorphous structures through hydrogelation from solutions with BMIMCl, followed by regeneration. Furthermore, we found that the produced materials exhibited a unique reswellable property, resulting in the reformation of hydrogels. The difference in the dissolution states of the cellulose chains, in accordance with the concentrations of the solutions, was also evaluated, which affected the characteristics of the hydrogels and porous cellulose.

## 2. Materials and Methods 

### 2.1. Materials

Absorbent cotton with an average degree of polymerization (DP) of 2000–5000 [1] was purchased from Hakujuji Corporation, Tokyo, Japan. An ionic liquid, BMIMCl (purity ≥98.0%), was purchased from Sigma-Aldrich, Darmstadt, Germany (catalog number 94128). All reagents were used as received.

### 2.2. Preparation of Cellulose Hydrogels 

Here, the experimental procedure was as follows (Entry 1, Table 1). A mixture of cotton cellulose (0.0250 g) with BMIMCl (0.503 g, previously dried at 100 °C for 3 h in a vacuum oven) was left standing at room temperature for 1 day and subsequently heated to 115 °C in a vacuum oven for 3 h, obtaining a 5 wt.% cellulose solution. The resulting solution was soaked in water (5.0 mL) for a day. The soaking procedures were additionally conducted twice (the total amount of water was 15 mL) to give a hydrogel. The resulting hydrogel was taken out from the aqueous media, washed with water several times, and wiped.

### 2.3. Preparation of Porous Celluloses and Their Reswelling Experiment

The hydrogel thus obtained was lyophilized for regeneration to produce a porous cellulose. The reswelling experiment was carried out by soaking the resulting porous cellulose in water (5.0 mL) for 2 days. The reformed hydrogel was taken out from the aqueous media, washed with water several times, and wiped.

### 2.4. Regeneration from Cellulose/BMIMCl Solutions Using Acetonitrile

Here, the experimental procedure was as follows. A 5 wt.% cellulose/BMIMCl solution (0.0506/0.100 (g/g)) was added with acetonitrile (20.0 mL) and the mixture was vigorously stirred for 30 min for the prompt regeneration of cellulose. The resulting regenerated cellulose was isolated by filtration, washed with water, and lyophilized.

### 2.5. Measurement

SEM images were obtained using a Hitachi S-4100H electron microscope (Hitachi High-Technologies Corporation, Tokyo, Japan), applying a 5 kV accelerating voltage. XRD profiles were obtained using a Rigaku Geigerflex RADIIB diffractometer (PANalytical B.V., EA Almelo, The Netherlands) with Ni-filtered CuKα radiation (*λ* = 0.15418 nm). Segal’s crystallinity indexes (CIs) were calculated from the peak intensity of the XRD patterns, and were were obtained from the following formulae [26,27,28]:

CI for cellulose I = (I_200_−I_18.5_)/ I_200_

CI for cellulose II = (I_110_−I_15.0_)/ I_110_.

The stress–strain curves were measured using rectangular strips (10 mm × 5 mm) of ca. 0.2 mm thickness on a tensile tester (Little Senstar LSC-1/30, Tokyo Testing Machine Co., Tokyo, Japan) with a cross head speed of 2 mm min^−1^.

## 3. Results and Discussion

**Figure 1:**
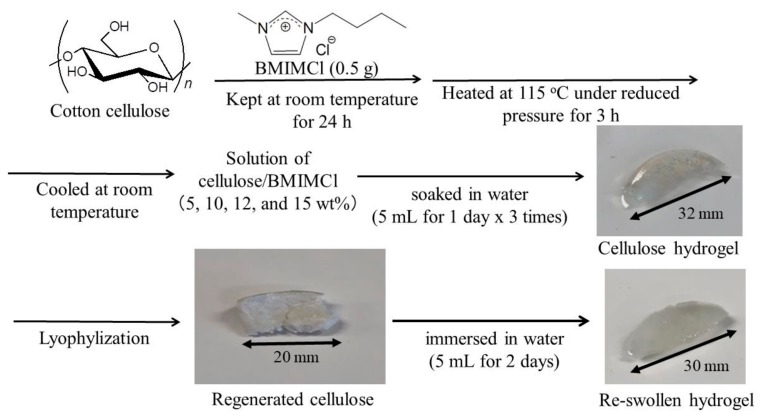
Procedure for the fabrication of amorphous porous celluloses through hydrogelation from solutions with 1-butyl-3-methylimidazolium chloride (BMIMCl) and their reswelling experiment.

As previously reported, a cellulose solution with BMIMCl can be facilely converted into a hydrogel by soaking it in water [25]. Therefore, in this study, four cellulose hydrogels were prepared from cellulose/BMIMCl solutions in different concentrations (5, 10, 12, and 15 wt.%) according to the procedure in Figure 1. The hydrogels were then lyophilized to produce the regenerated celluloses. The water contents of the hydrogels were calculated by weight differences before and after lyophilization. As listed in Table 1, the water contents decreased with increasing the cellulose concentrations in the initial solutions with BMIMCl. Based on the weight differences between the regenerated celluloses and the starting celluloses, we also confirmed that BMIMCl was mostly not present in the hydrogels (Table 1, 0.38–0.68 % in a total amount of BMIMCl). Similar characteristics of the hydrogels were found after repeating the procedures several times.

**Figure 2:**
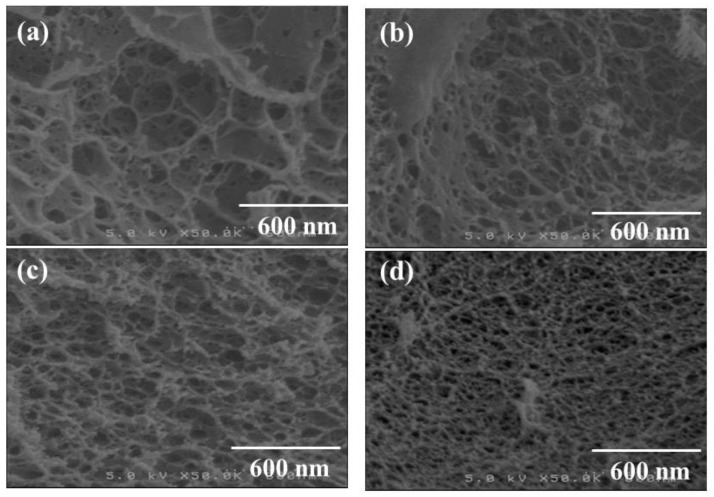
SEM images of regenerated celluloses obtained by the lyophilization of the hydrogels (**a**–**d**, Entries 1–4).

**Figure 3:**
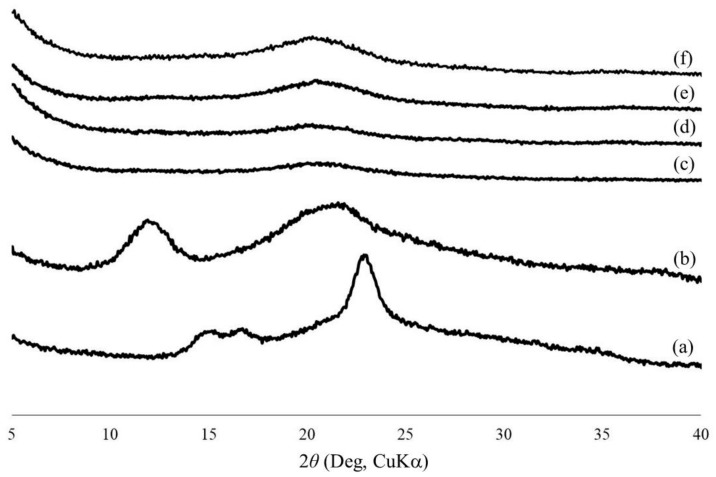
XRD profiles of cotton (cellulose I) (**a**), cellophane (cellulose II) (**b**), and the regenerated celluloses obtained by the lyophilization of the hydrogels (**c**–**f**, Entries 1–4).

**Figure 4:**
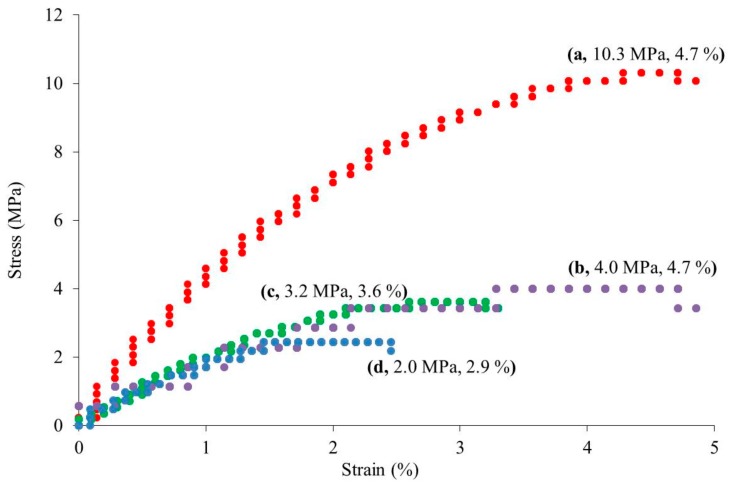
Stress–strain curves of the regenerated celluloses obtained by the lyophilization of the hydrogels (**a**–**d**, Entries 1-4) under a tensile mode. Values of tensile strength and elongation at break are shown in parentheses.

The morphologies of the regenerated celluloses were evaluated by SEM measurements (Figure 2). The SEM images of all the samples exhibit porous morphologies, where the pore sizes obviously decrease with the increase in concentration of the initial solutions with BMIMCl. Here, the pore sizes were probably dependent on the network sizes of the cellulose chains in the hydrogels. Therefore, it can be reasonably explained that the difference in the network sizes, or in turn the pore sizes, as observed in the SEM images, affected the water contents of the hydrogels, in which a larger network exhibits an ability to absorb a larger amount of water, as shown in Table 1. The crystalline structures of the porous celluloses were then evaluated via XRD measurement. Figure 3 shows the XRD profiles of the regenerated porous celluloses of Entries 1–4, in comparison with those of cotton with a cellulose I crystalline structure and cellophane with a cellulose II crystalline structure. All the XRD profiles of the porous celluloses did not exhibit obvious diffraction peaks assignable to either the cellulose I or cellulose II crystalline structures, strongly indicating the amorphous structures of the porous celluloses. The mechanical properties of the porous celluloses were investigated by tensile testing. The resulting stress–strain curves (Figure 4) indicate an enhancement in both tensile strength and elongation at break, in accordance with the increase in pore size, which is dependent on the concentration of the initial solution with BMIMCl. The results of tensile testing thus suggest that the porous cellulose, with a small pore size showing a brittle nature (Entry 4, Figure 4d), and the elongation values at break increasing (Figure 4a–c) with the increase of pore size (Entries 1–3). In addition to the large elongation value at break, the porous cellulose with the large pore size (Entry 1, Figure 4a) particularly exhibited a much better tensile strength than that of the other materials. The porous cellulose with a small pore size (Entry 4) showed similar mechanical properties to that of the general regenerated cellulose, which has a brittle nature. The increase in the pore size provided the toughness of the materials, contributing to enhancement of both the tensile strength and the elongation at break. The repetitions of tensile testing gave similar stress–strain curves, as shown in Figure 4.

**Figure 5:**
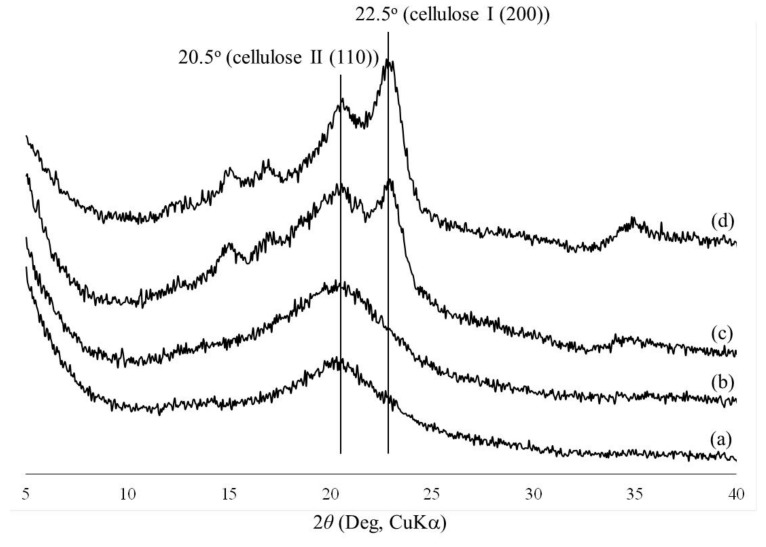
XRD profiles of regenerated celluloses from cellulose/BMIMCl solutions (**a**–**d**, 5, 10, 12, and 15 wt.%) using acetonitrile.

The effect of the concentration of the cellulose/BMIMCl solution on the network sizes of cellulose chains in hydrogels (or pore sizes in the porous celluloses) has been proposed to be in accordance with the dissolution states of cellulose chains in BMIMCl. We found that cellulose could be promptly regenerated by adding acetonitrile in cellulose/BMIMCl solutions, followed by vigorously stirring the mixtures, as the crystalline structures of the regenerated celluloses reflected the dissolution states in accordance with the concentrations. The XRD profiles of the samples regenerated from the 5 and 10 wt.% solutions using acetonitrile show broad peak patterns assignable to a cellulose II crystalline structure, with CI values of 27.2 and 36.4 %, respectively (Figure 5a,b). On the other hand, the XRD profiles of the other samples regenerated from the 12 and 15 wt.% solutions exhibit obvious diffraction patterns assignable to both the cellulose I and cellulose II crystalline structures (Figure 5c,d). The CI values of the latter sample (45.6 (I) and 30.1 (II) %) were higher than those of the former sample (24.0 (I) and 22.2 (II) %) in both the cellulose I and cellulose II. Furthermore, the CI value of the sample regenerated from the 15 wt.% solution for cellulose I was higher than that of cellulose II, while the values of the sample regenerated from the 12 wt.% for cellulose I and cellulose II were comparable. The remaining cellulose I crystalline structure in these regenerated samples strongly indicates that the dissolution states of cellulose chains in the solutions in such concentrations are not under typical molecular dispersion conditions, because cellulose I has been well-known to transform into cellulose II through dissolution with molecular dispersion. Furthermore, the higher CI values of the cellulose I crystalline structure were confirmed to be in accordance with the increase of concentration in the solutions (24.0% (12 wt.%) and (45.6 % (15 wt.%)). The previous study also reported that the cellulose I crystalline structure partially remained, according to the conditions of the dissolution of cellulose with BMIMCl [29]. These XRD results and CI values strongly support that the dissolution states of the cellulose chains in solutions with lower concentrations were relatively under molecular dispersion conditions. In higher concentrations, the cellulose chains interacted more tightly, resulting in smaller network sizes in the hydrogels, which also gave rise to the construction of smaller pore sizes in the regenerated celluloses by lyophilization.

Finally, we examined the reswelling of the amorphous porous celluloses through immersion in water for 2 days (Figure 1). Consequently, all the materials reswelled to regenerate hydrogels. The water contents of the produced hydrogels were slightly lower than those of the primary prepared hydrogels shown in Table 1. In addition to porous morphologies, amorphous structures probably contributed to enhancing water absorption among the cellulose chains, contributing to the exhibited reswellable property of the present materials.

## 4. Conclusions

In this study, the reswellable amorphous porous celluloses were successfully fabricated via regeneration from hydrogels, which were prepared from solutions in BMIMCl. The resulting porous celluloses were characterized by SEM, XRD, and tensile testing measurements. The concentrations of the solutions strongly affected the pore sizes and mechanical properties of the materials. These observations were representatively found to result from a difference in network size in the hydrogels, reflected by the solution states of cellulose chains depending on the concentrations. Furthermore, the porous celluloses showed a reswellable property after immersing them in water, reforming the hydrogels. Such a unique property provides suitability for the use of the cellulosic materials for biomedical and drug delivery applications. The present cellulosic materials also have potential to be practically used in bio-based and eco-friendly application fields in the future. Furthermore, the present approach will be applied to fabricate additional cellulosic materials via gelation with different dispersion media from water.

## Figures and Tables

**Table 1:** Water contents of hydrogels and the residual BMIMCl amounts in porous celluloses.

Entry	Cellulose Concentration in BMIMCl Solution (wt.%)	Water Content of Primary Hydrogel (wt.%) ^(a)^	Residual BMIMCl in Porous Cellulose (wt.%) ^(b)^	Water Content of Reswollen Hydrogel (wt.%) ^(a)^
1	5	94.8	0.52	90.8
2	10	90.7	0.60	85.2
3	12	89.3	0.68	82.2
4	15	85.4	0.38	69.6

^(a)^ Estimated by the weight differences between the hydrogels and regenerated celluloses. ^(b)^ Estimated by the weight differences between the regenerated celluloses and starting celluloses.
